# 

**Published:** 1916-01

**Authors:** 


### CONTENTS, JANUARY, 1916—VOLUME XXXI, NO. 7.

### ORIGINAL ARTICLES.

Hemoptysis—Report of Three Cases Treated by Artificial Pneumo-Thorax. By Alvis E. Greer, M. D., Houston, Texas........... 265
The New Discovery About Pneumonia. By Dr. Leonard Keene Hirshberg, A. B., M. A., M. D., Johns Hopkins................269

### DEPARTMENT OF EUGENICS.

Mental Efficiency ............................................ 273
Eugenic and Euthenic Notes.................................. 275
Value of Negative Eugenics. By Edwin G. Conklin, Professor of Biology, Princeton University, Princeton, N. J............... 276

### EDITORIAL DEPARTMENT.

The New Year.................................................. 282
Typhoid Responsibility. By Dr. G. Henri Bogart, Shelbyville, Ill.. 286

Items of General Interest.................................... 287
Marvels of Surgery Performed by Russians.................... 291
Doctors Believe Extermination of Malaria Possible.............. 292
Public Health Report of the Secretary of the Treasury.......... 293
Personal Mention ............................................ 294
Medical Meetings ............................................ 295
Deaths ........................................................ 298
Book Reviews ................................................ 299
Helpful Hints for the Busy Doctor........................... 302

